# Aptamer-targeted cell-specific RNA interference

**DOI:** 10.1186/1758-907X-1-4

**Published:** 2010-02-01

**Authors:** Jiehua Zhou, John J Rossi

**Affiliations:** 1 Division of Molecular and Cellular Biology, Beckman Research Institute of City of Hope, City of Hope, Duarte, CA 91010, USA; 2 Irell and Manella Graduate School of Biological Sciences, Beckman Research Institute of City of Hope, City of Hope, Duarte, CA 91010, USA

## Abstract

This potent ability of small interfering (si)RNAs to inhibit the expression of complementary RNA transcripts is being exploited as a new class of therapeutics for a variety of diseases. However, the efficient and safe delivery of siRNAs into specific cell populations is still the principal challenge in the clinical development of RNAi therapeutics. With the increasing enthusiasm for developing targeted delivery vehicles, nucleic acid-based aptamers targeting cell surface proteins are being explored as promising delivery vehicles to target a distinct disease or tissue in a cell-type-specific manner. The aptamer-based delivery of siRNAs can often enhance the therapeutic efficacy and reduce the unwanted off-target effects of siRNAs. In particular, for RNA interference-based therapeutics, aptamers represent an efficient agent for cell type-specific, systemic delivery of these oligonucleotides. In this review, we summarize recent attractive developments in creatively using cell-internalizing aptamers to deliver siRNAs to target cells. The optimization and improvement of aptamer-targeted siRNAs for clinical translation are further highlighted.

## Introduction

RNA interference (RNAi) refers to the sequence-specific cleavage of messenger RNA that follows the cellular introduction of complementary, small interfering (si)RNA duplexes 21 to 25 nt in length [1,2]. The development of siRNA-based therapeutics has progressed rapidly because of their specific and potent RNAi triggering activity [3,4]. Although siRNAs offer several advantages as potential new bio-drugs to treat various diseases [4-6] including cancers and HIV infection [7], the efficient delivery of siRNAs *in vivo *remains a crucial challenge for achieving the desired RNAi effect in clinical development [5,8,9].

In particular, a targeted intracellular delivery approach for siRNAs to specific cell populations or tissues is highly desirable for the safety and efficacy of RNAi-based therapeutics. Targeted delivery of therapeutics is an area of vigorous research, and numerous recent investigations have described cell type-specific siRNA delivery using different strategies. For example, siRNAs have been covalently conjugated to a targeting ligand (cholesterol [10], α-tocopherol [11], lipophilic molecule [12,13], short peptide and antibody [14,15], agonist molecule [16] and nucleic acid-based aptamer [17-20]). Alternatively, siRNAs have also been non-covalently assembled with active recognition moieties and delivery vehicles as multifunctional targeting delivery systems, such as folate conjugated dendrimer [21], folate-conjugated phage RNAs [22-24], transferrin modified polymer/liposomes [25-28], peptide-based nanovectors [29-32], cholesterol polymers [33], antibody-mediated delivery formulations [34-48] and aptamer delivery platforms [20,49-51]. The last holds great promise for clinical translation. An ideal targeted delivery system contains two essential elements: (i) a potent therapeutic siRNA and (ii) a targeting vehicle that can selectively recognize and effectively escort cargo into a particular organ or cell. Indeed, a targeting ligand with high specificity and affinity to a cellular receptor is a major factor in establishing a targeted siRNA delivery system.

Nucleic acid-based aptamers offer some important features for targeted siRNA delivery [52-55]. Aptamers are *in vitro *selected nucleic acids that assume specific and stable three-dimensional shapes, thereby providing highly specific, tight binding to targeted molecules [56-58]. Given a specific molecular target, aptamers can be identified from combinatorial libraries of nucleic acids by a technique called systematic evolution of ligands by exponential enrichment (SELEX) [57]. Despite the relative youth of the aptamer field, nucleic-acid aptamers have extensively blossomed in various fields ranging from diagnostics to therapeutics [59-61]. In particular, a new concept known as 'escort aptamers', a term first used by Hicke and Stephens [52] suggests a new facet of aptamer functionality: aptamers as drug-delivery devices. Aptamers, also described as nucleic acid versions of antibodies, possess some unique characteristics that derive from their nucleic acid composition (for example, lack of immunogenicity *in vivo*, relatively small physical size, straightforward chemical synthesis that makes them amenable to backbone modification, and rapid *in vitro *selection), making them more adaptable for specifically delivering a variety of reagents to targeted cells or tissues [59]. Moreover, precise site-specific modifications facilitate engineering of aptamers for this special purpose.

Currently, a number of aptamers targeting specific cell surface receptors have been successfully adapted for the targeted delivery of active drug substances both *in vitro *and *in vivo*, including anti-cancer drugs [53,62-72], toxins [73], enzymes [74], radionuclides [75], virus [76] and siRNAs [17-20,22,49,77] (Table 1). The cargoes are attached to the aptamers either through direct conjugation to the aptamer or through their assembly with functionalized groups appended to the aptamer and cargos. As anticipated, aptamer-mediated targeted delivery can enhance the therapeutic efficacy and reduce the toxic effects of drugs. For example, Neufeld and colleagues successfully delivered the enzyme α-L-iduronidase to the lysosomes of cells deficient in this enzyme using aptamers targeted to the mouse transferrin receptor (TfR) [74]. For RNAi-based therapeutics, several groups have applied cell-internalizing aptamers to specifically deliver siRNAs to target cells. The most established and best characterized aptamers for siRNA delivery are the prostate-specific membrane antigen (PSMA) aptamers that bind with high affinity to PSMA [78]. Three separate groups [18,49,77] have constructed distinct aptamer-siRNA conjugates for successful delivery of siRNAs into tumor cells. Functional optimization of these conjugates has been carried out [17,77] (for example, truncation or multimerization of aptamers, enhanced loading efficiency and stability of the siRNAs, and various aptamer-siRNA linkage designs and conjugation approaches), making aptamer-mediated RNAi therapeutics a promising approach for future clinical translation. There is increasing enthusiasm for generating new, more potent cell-internalizing aptamers and for developing novel and rapid selection strategies (such as cell-based SELEX [79-82] and automated SELEX workstations [83-86]) to exploit the clinical potential of aptamer-mediated delivery systems. This review focuses on recent progress in aptamer-mediated siRNA delivery for treatment of human diseases.

**Table 1:** Cell-internalizing aptamers for targeted delivery.

Cell-internalizing aptamers	Cargoes and strategy for targeted delivery
RNA aptamers against PSMA	1) siRNA (non-covalently conjugate siRNA with aptamer via a streptavidin connector [49]; aptamer-siRNA chimeras [17,18] and bivalent aptamer-siRNA conjugates [77]).
	
	2) Toxin [73] (chemically covalently conjugate toxin with aptamer via SPDP reagent)
	
	3) Nanoparticles and chemotherapeutic agents [62-70] (cargoes such as dextran, docetaxel, Pt(IV) and doxorubicin were encapsulated into aptamer-coated nanoparticles; aptamer-Dox physical conjugates via intercalation interaction)

RNA aptamers against CD4	siRNA [25] (non-covalently assemble pRNA-siRNA chimera with pRNA-aptamer chimera into dimer or trimer)

RNA aptamers against HIV gp120	siRNA [19,20] (aptamer-siRNA chimeras; non-covalently conjugate siRNA with aptamer via a 'sticky bridge')

RNA aptamers against TN-C	Radionuclide and fluorescent agents (chemically covalently conjugate ^99m^Tc or fluorescent agents with aptamers)

DNA aptamers against PTK7	1) Doxorubicin [93] (chemically covalently conjugate Dox with aptamer via an acid-labile linkage)
	
	2) Viral capsid [94] (chemically covalently conjugate MS2 viral capsid with aptamer via an oxidative coupling reaction)

DNA aptamers against TfR	Enzyme [74] (chemically covalently conjugate a-L-iduronidase with aptamer via an oxidative coupling reaction)

DNA aptamers against NCL	Liposomes and chemotherapeutic agents [71] (cisplatin was encapsulated into liposomes that was non-covalently coated with aptamers)

DNA aptamers against MUC1	Photodynamic therapy agents [72] (chemically covalently conjugate chlorine e6 with aptamer via EDC chemistry)

The RNA or DNA aptamers used as delivery vehicles for various cargoes via different strategies are listed in the table.

EDC, (1-ethyl-3-[3-dimethylaminopropyl]carbodiimide hydrochloride); gp120, glycoprotein 120 (envelope protein); MUC1, mucin protein (membrane-associated glycoprotein); NCL, nucleolin (a bcl-2 mRNA binding protein); pRNA, phage RNA; PSMA, prostate-specific membrane antigen; PTK7, protein tyrosine kinase 7 (a transmembrane receptor); siRNA, small interfering RNA; SPDP, N-[O-succinimdy]-3-(2-pyridyldithio) propionate; TfR, Transferrin receptor (in mouse); TN-C, tenasin-C (a hexameric glycoprotein).

## Development of cell-internalizing aptamers

Efficient development of new cell-type specific internalizing aptamers presents a major challenge because of the limited number of purified receptors that can be used for aptamer selection when the protein targets are insoluble or the targets are functionally part of multiprotein complexes. In these situations, traditional purified protein-based *in vitro *selection is not feasible. Therefore, protocols based on live cell selection present an alternative method for identifying aptamers against either cell surface or cell internal proteins. In contrast to the purified protein-based SELEX method, cell-based SELEX [79] can be performed even with unknown targets or multiprotein complexes expressed on the cell surface. Moreover, because intact living cells with many native receptor proteins are used as targets during the selection procedure, panels of new aptamers can be isolated from such screens [80]. Because this strategy relies essentially on the differences between the target cell population with particular features relative to the control cell population used for counterselection, (for example: defined phenotype, protein expression level, different protein conformations), multiple binding species that recognize only the target cells and not the control cells can be identified.

Despite these advantages, it should be noted that this approach does not discriminate between dead cells with reduced cell-membrane integrity and cells that are living [60]. Because dead cells can yield a sequence-independent binding of nucleic acids, cell-based SELEX can be inefficient for aptamer selection [87]. During the process of treating cells with the SELEX libraries, any damage to fragile cells might incur the risk of selection failure. Compared with the traditional SELEX methods using a single target protein [88], cell-based SELEX usually requires more selection cycles (>20) and longer processing times for efficient enrichment of an aptamer population. Furthermore, increasing the number of selection cycles often favors the enrichment of nonspecific or unwanted species, which preferentially adapt to the enzymatic amplification reactions rather than to the target binding. These facts therefore demonstrate that aptamer selection involving living cells is a difficult task and is still in its infancy. Although successful in individual cases [80], further optimization of the selection schemes is required to increase the general applicability. For example, living and dead cells within a cultured cell mixture could be discriminated and separated on the basis of their different light-scattering characteristics [87].

As reported recently, several cell-internalizing aptamers against cell surface biomarkers or receptors have been successfully selected as targeting vehicles, through either traditional recombinant protein-based SELEX or cell-based SELEX strategies. To date, it has been demonstrated that RNA aptamers against PSMA [78], CD4 [89], HIV glycoprotein 120 [20,90,91] and tenascin-C protein (TN-C) [92], and DNA aptamers against protein tyrosine kinase-7 (PTK7) [93,94], mouse Transferrin Receptor (TfR) [74], nucleolin (NCL) [71] and mucin 1 (MUC1) [72], can be used for targeted delivery purposes (Table 1). Despite these validated examples, there is still a need for additional potent cell-internalizing aptamers to expand the diversity of targeting ligands and promote their potential therapeutic applications.

## Aptamer-mediated cell-type specific siRNA delivery

Cell-internalizing aptamers are well suited to cell type- or tissue-specific delivery of various cargoes because of their high affinity and specificity, and their accessibility for backbone modifications. Approaches in which aptamers and siRNAs have been linked to achieve targeted siRNA delivery and enhance RNAi potency, and to reduce unwanted side-effects have recently been described. Therefore, this section will focus on the aptamer-mediated siRNA delivery approaches. So far, only three RNA aptamers have been exploited for this purpose; however, many other aptamers such as those listed in Table 1 may also be useful for targeted siRNA delivery.

### Anti-PSMA RNA aptamer-mediated RNAi

PSMA is a well-characterized transmembrane protein, which is strongly expressed in human prostate cancer and the vascular endothelium [95,96]. Importantly, PSMA is continually recycled from the plasma membrane and is constitutively endocytosed in PSMA-positive LNCaP cells, making it an attractive portal to deliver molecules intracellularly [66]. An anti-PSMA monoclonal antibody was demonstrated to promote the internalization rate. Using a purified fusion target protein containing a modified extracellular form of PSMA, Lupold *et al*. previously selected from an RNA library two 2'-fluoro (2'-F)-modified RNase-resistant RNA aptamers (A-9 and A-10) with low nanomolar affinity binding constants [78]. They also quantified the affinity of each aptamer for PSMA by measuring the inhibition of N-acetylated α-linked acidic dipeptidase (NAALADase) activity. Aptamer A-9 inhibited PSMA noncompetitively with an average K_i _of 2.1 nM, whereas aptamer A-10 inhibited competitively with an average K_i _of 11.9 nM. Because these anti-PSMA aptamers can be internalized, they have recently been engineered for cell-type specific delivery of various cargoes [53,54,61], such as chemotherapeutic agents, drug-encapsulated nanoparticles, toxins, enzymes and siRNAs.

**Figure 1:**
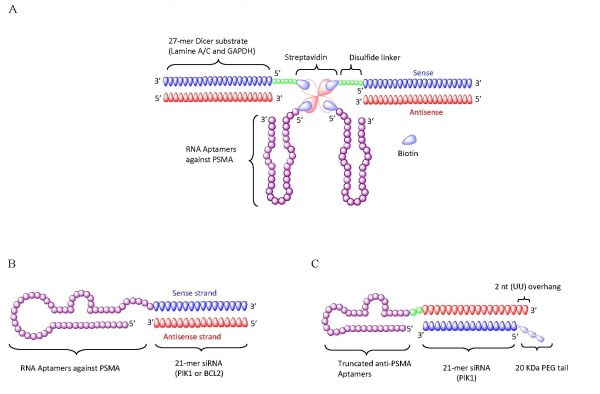
**Anti-prostate-specific membrane antigen (PSMA) aptamer-mediated small interfering (si)RNA delivery**. (a) Schematic of anti-PSMA aptamer-streptavidin-siRNA conjugates. The 27-mer Dicer substrate RNA duplex and RNA aptamers were chemically conjugated with a biotin group. Thus, two biotinylated siRNAs and two aptamers were non-covalently assembled via a streptavidin platform. (b) Schematic of the first generation anti-PSMA aptamer-siRNA chimeras. The 2'-Fluoro-modified aptamer and siRNA sense strand were co-transcribed, followed by annealing of the complementary siRNA antisense strand to complete the chimeric molecule. **(c) **Schematic of the optimized second generation chimeras. Compared with the first generation chimeras, the aptamer portion of the chimera was truncated from 71 to 39 nucleotides, and the sense and antisense strands of the siRNA portion were swapped. A 2 nucleotide (UU)-overhang and a polyethylene glycol tail were added to the 3'-end of the guide strand and the 5'-end of passenger strand, respectively.

Three independent groups have successfully employed the anti-PSMA RNA aptamers to specifically deliver siRNAs to target cells (Figure 1). In a proof of concept study [49], Chu *et al*. recently reported successful non-covalent conjugation of biotinylated anti-PSMA aptamer (A-9) with biotinylated 27-mer lamin A/C or GAPDH siRNAs via a modular streptavidin connector (Figure 1a). To enhance siRNA release in the cytoplasm, a reducible disulfide linker was designed between the sense strand of siRNA and the biotin group. By using such a streptavidin connector, two aptamers and two siRNAs were elegantly assembled into a multivalent construct, displaying effective PSMA receptor-mediated internalization of aptamer-siRNAs and specific silencing of the targeted transcripts in tumor cells.

A somewhat different approach was developed by Giangrande and colleagues [18], in which a 2'-F-modified anti-PSMA aptamer (A-10) was covalently appended to the sense strand of a 21-mer siRNA portion, which in turn was hybridized to the 21-mer antisense strand (Figure 1b). The resulting aptamer-siRNA chimeric RNA was shown to be selectively internalized into cells expressing PSMA, and to effectively knock down expression of the targeted the tumor survival genes (PLK1 and BCL2) both in cell culture and *in vivo *after intratumoral delivery. Because this delivery system consists only of RNA components, it offers several potential advantages as a therapeutic agent, including lack of immunogenicity, the possibility for chemical synthesis, and stabilizing modifications for *in vivo *application.

Although aptamer-siRNA chimeras can be directly administered to localized targets (for example, intratumoral delivery), systemic administration will be required for many diseases. In general, greater therapeutic doses are required for systemic administration, leading to higher costs and side effects. Most recently, Giangrande and colleagues addressed this issue by optimizing their previous PSMA-siRNA chimeric design to achieve enhanced inhibition of prostate cancer xenograft growth via systemic administration (Figure 1c) [17]. First, the aptamer portion of the PSMA A10-Plk1 chimera was truncated from 71 to 39 nucleotides, while still maintaining high binding affinity. Most importantly, the truncated version (containing the aptamer and sense strand of the siRNA) comprising a total of 64 nucleotides makes this amenable to chemical synthesis. Second, the silencing potency was enhanced through structural modifications of the siRNA portion, enabling more efficient incorporation of the siRNA by the cellular RNAi machinery. The group then added a 2-nucleotide (UU) overhang at the 3' end of the siRNA duplex, but also swapped the positions of the passenger and guide strands of the siRNA. These modifications favor Dicer recognition and loading of the guide strand (containing the two base 3' overhang) into an RNA-induced silencing complex (RISC), hence increasing the silencing activity and specificity. By appending a polyethylene glycol moiety with molecular weight 20 kDa onto the siRNA passenger, the circulating half-life of the chimeric molecule was substantially increased and the bioavailability was markedly improved, leading to prolonged silencing *in vivo*. As a result of these efforts, the optimized second-generation aptamer-siRNA chimeras (Figure 1c) resulted in pronounced regression of PSMA-expressing tumors after systemic administration in athymic mice. Additionally, the therapeutic dose of the new chimera was dramatically reduced from 1 nmol on each of 10 successive days (10 × 1 nmol) to 0.25 nmol in every other day for a total of 10 days (5 × 0.25 nmol), minimizing both the cost of treatment and the risk of harmful side effects.

Other efforts to further refine aptamer-mediated siRNA delivery and targeting efficiency are being attempted through multimerization of the aptamer portion. Previous studies with aptamers have revealed that multivalent versions of aptamers can increase the potency and antitumor response, and promote receptor activation [97-100]. The multivalent aptamer-siRNA construct has also been recently exploited for facilitating receptor internalization, further improving the therapeutic potential. Wullner *et al*. generated two different bivalent anti-PSMA aptamer-siRNA chimeras in which the siRNAs targeted eukaryotic elongation factor 2 [77]. Their modifications included using the siRNA itself as a linker to join the two aptamers or appending the siRNAs onto the 3' ends of each aptamer. Compared with the monovalent aptamer-siRNA chimeras (55% target knockdown), these bivalent aptamer-siRNA constructs resulted in an almost complete loss of PSMA-positive cell viability, suggesting that bivalent aptamers definitely promote internalization of chimeras. These efforts have encouraged new thinking in the design of multiple aptamer-siRNA conjugates.

### Anti-CD4 RNA aptamer-mediated RNAi

The CD4 receptor, a glycoprotein expressed on the surface of certain subsets of T lymphocytes [101-103], is a primary receptor used by HIV-1 to gain entry into host T cells. It was previously reported that overexpressed CD4 protein in T helper cells can be endocytosed [104]. Aptamers targeting CD4 were produced by immobilizing soluble, recombinant CD4 antigen onto Sepharose beads, allowing elution of unbound oligonucleotides and retention of bound species, which were further amplified for the next selection rounds [89]. Using this approach, 2'-F-modified RNA aptamers with high CD4 affinity were identified. The ability of CD4 aptamers to block functional T cell responses was tested using an allogeneic mixed lymphocyte reaction (MLR), a complex *in vitro *assay of T-cell recognition and responsiveness, in which the comparative standard is the W3/25 CD4 monoclonal antibody, which binds to the same site as the tested aptamer clones. Thus, the ability to block MLR correlates with CD4 binding activity. These aptamers showed inhibitory effects in a CD4-specific manner. This CD4-specific aptamer has been assembled into a multifunctional nano-device for targeted delivery of siRNAs in a T-cell line engineered to overexpress CD4 [22,50].

**Figure 2:**
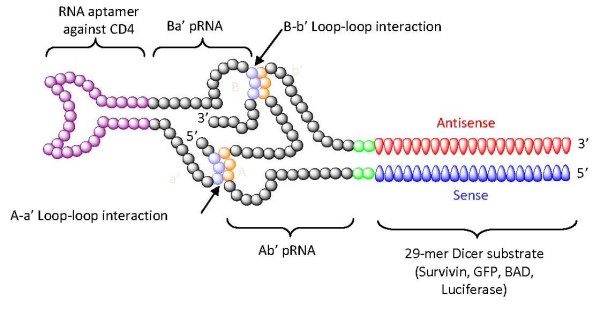
**Anti-CD4 aptamer mediated small interfering (si)RNA delivery**. Schematic of a dimer of the chimeric phage (p)RNA-CD4 aptamer and chimeric pRNA-siRNA. The anti-CD4 aptamer or siRNAs were non-covalently joined via phi29 RNAs containing complementary loop domains. Through interactions of the interlocking left and right loops, chimeric phi29 RNAs could be fabricated into the dimers shown as an example.

Recently, anti-CD4 RNA aptamers have been exploited for targeted delivery of siRNAs [22,105]. The self-assembling bacteriophage phi29 RNA (pRNA) was joined with the anti-CD4 aptamer and allowed to form a nano-complex with a pRNA-siRNA chimera. It was previously demonstrated that pRNAs can be accurately assembled through interlocking right- and left-hand loops into various oligomers (dimer, trimer, hexamer) ranging in size from nanometers to micrometers [51,106]. The pRNA itself can be fused with various agents (folate, aptamer, siRNA, dye, antitumor drugs), while still allowing oligomerization of the pRNAs [107]. For example, as shown in Figure 2, two pRNA molecules were respectively fused with siRNAs (against survivin, green fluorescent protein (GFP), Bcl2 antagonist of cell death (BAD) or luciferase) and the anti-CD4 aptamer. Through the interaction of right and left interlocking loops, the two chimeric pRNAs could be precisely dimerized into a stable nanovector of approximately 25 nm in diameter. The nano-scale RNA dimer was also shown to be effectively internalized into a CD4-overexpressing T cell line, and the siRNAs consequently knocked down the expression levels of the targeted surviving or enhanced GFP mRNAs. The direct correlation between CD4 expression level and the internalization/silencing activity of the siRNAs also provided proof of anti-CD4 aptamer-mediated cell-specific siRNA delivery. Similarly, a trimeric conjugate was engineered in the same way. Three chimeric pRNA building blocks (one fused with the CD4 aptamer, another with an siRNA and a third with a fluorescent molecule) were assembled into a multifunctional nano-device, which elicited siRNA-mediated target knockdown and also provided molecular imaging via the fluorescent dye. This self-assembling nano device may improve the *in vivo *kinetics and enhance the therapeutic efficacies of the delivered siRNAs.

### Anti-gp120 RNA aptamer-mediated RNAi

The HIV-1-encoded gp120 protein, a glycoprotein envelope on the surface of HIV-1, plays an important role in viral entry into CD4 cells [101,103]. The interaction of gp120 and CD4 triggers HIV-1 entry and initiates cell fusion [108-111]. Recently, a chimeric Fab gp120 antibody fragment-protamine fusion was demonstrated to facilitate receptor-specific siRNA uptake into cells expressing the HIV-1 gp120 envelope protein, suggesting this protein as a new molecular target for receptor-mediated siRNA delivery [40]. Several 2'-F modified anti-HIV gp120 RNA aptamers have been isolated with the use of a BIAcore biosensor system (Stevenage, United Kingdom) [90,91,112,113] or conventional nitrocellulose filter binding of aptamers to recombinant proteins [20]. The selected aptamers can specifically bind to and be rapidly internalized into cells expressing the HIV-1 envelope protein. In addition, the aptamers alone can neutralize HIV-1 infectivity.

**Figure 3:**
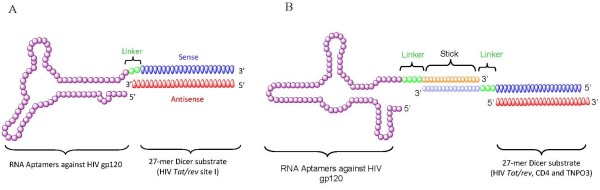
**Anti-HIV-1 gp120 aptamer-mediated small interfering (si)RNA delivery**. (a) Schematic of the anti-HIV-1 gp120 aptamer-siRNA chimeras. The anti-gp120 aptamer binds to gp120 and the 27-mer Dicer substrate RNA duplex targets a common exon of the HIV-1 *tat/rev *transcript. Dicer processing results in 21-mer siRNAs that are incorporated into an RNA-induced silencing complex (RISC). (b) Schematic of the anti-HIV gp120 aptamer-'sticky bridge'-siRNA conjugates. Either the antisense or the sense strand of the 27-mer Dicer substrate RNA duplex and the aptamer were attached with to complementary 'sticky' sequences. After a simple annealing, they form stable base pairs.

Recently, we used gp120 aptamer-siRNA chimeras for cell type-specific delivery of siRNAs in cultured cells and in a HIV-1 infected Rag-Hu mouse model. The gp-120 aptamer (Figure 3a), was covalently linked to siRNAs that target the HIV-1 *tat/rev *common exon [19]. Because both the aptamer and the siRNA can inhibit HIV-1 replication by respectively blocking the gp120-CD4 receptor interaction and silencing HIV-1 *tat/rev *expression, this novel anti-gp120 aptamer-siRNA chimera possesses a dual inhibitory function. Treatment of HIV-1-infected cells with these chimeras resulted in the selective gp120-mediated internalization of the aptamer-siRNA by endocytosis and the specific silencing of the targeted mRNA transcript. Interestingly, a small change in the length of the siRNA portion of the chimera from 21bp to 27bp resulted in enhanced silencing potency. This was the result of Dicer processing of the 27-mer from the aptamer and perhaps a more efficient handoff of the processed siRNA to RISC. These results demonstrated that HIV-1 gp120 expressed on the surface of HIV-1-infected cells represents a unique target for aptamer-mediated siRNA delivery.

In a study by Zhou *et al. *[20], a 'sticky bridge' strategy was developed to non-covalently conjugate the aptamer with various siRNAs (Figure 3b). In this design format, one pair of complementary GC-rich sticky bridge sequences was chemically attached to the 3' end of the aptamer. The complement to this sequence was attached to one of the two siRNA, strands and the aptamer and siRNA were joined by Watson-Crick base pairing. A flexible three-carbon atom hinge (C3) was added as a spacer between the adhesive (sticky) sequence and the aptamer to allow spatial and structural flexibility. Importantly, this sticky bridge-based strategy can be used to facilitate the effective interchange of different siRNAs with a single aptamer, which is required to avert viral resistance to the siRNA component. We combined three different siRNAs with the gp120 aptamer: one against the HIV-1 *tat/rev *gene, and two siRNAs targeting the HIV host dependency factors CD4 and transportin 3, respectively. The specific binding and internalization of the aptamer-siRNA conjugates into gp120-expressing cells was demonstrated by confocal microscopy, and the aptamer-'sticky bridge'-siRNA combinations downregulated targeted gene expression and suppressed HIV replication in cell culture. Additionally, the aptamer-siRNA combinations also served as dual-function inhibitors, providing additive efficacy. These results demonstrated the potential use of aptamer-siRNA conjugates as a systemic, cell type-specific, siRNA cocktail delivery system for anti-HIV-1 therapy.

Most recently, we tested the anti-HIV efficacy of these aptamer-siRNA dual inhibitors in a humanized mouse model (P. Neff *et al*., manuscript submitted to Science Translational Medicine). In this model system, the humanized Rag2^-/-^γc^-/- ^mice (RAG-hu) were treated with human CD34 hematopoietic progenitor cells, which engraft and differentiate in a variety of human hematopoietic lineages. The mature T cells and monocytes were infected with the HIV-1 NL4.3 virus. After 3 weeks of viral replication, the animals were injected intravenously once weekly with the aptamer-siRNA conjugates. We observed a dramatic decrease in viral load in all the treated animals, in most cases to undetectable levels within a week after the intravenous administration of the chimera. The suppression of viral load averaged three logs of reduction relative to controls, and persisted throughout and beyond the treatment period in several of the animals. Most importantly, the aptamer-siRNA treatment completely prevented T-cell depletion mediated by viral infection. Therefore, the capacity to achieve marked viral suppression *in vivo *together with restoration of CD4 T cell levels using aptamer-siRNA constructs should pave the way for implementing novel therapeutic strategies for treating HIV disease. In particular, these dual-action constructs will be useful for treatment of patients who do not respond to highly active anti-retroviral therapy, the standard multi-drug treatment that has proved so effective in battling AIDS.

## Conclusions and perspectives

Since the first description of RNA interference triggered by double-stranded RNA in 1998, RNAi has rapidly become one of the methods of choice for gene function studies and is also extensively being exploited for therapeutic applications. The successful use of siRNAs for therapeutic purposes requires safe and efficient intracellular delivery to specific cells and tissues. Nucleic acid-based aptamers have many favorable characteristics, including high binding sensitivity and specificity, small size and ease of *in vitro *selection, making them very attractive for a variety of uses in molecular targeting. In this regard, nucleic acid aptamers targeting cell surface proteins are emerging as a promising class of delivery vehicles to target a particular cell population or tissue, thus providing enhanced therapeutic potency and reduced cellular toxicity.

To date, significant advances have been made to develop cell-internalizing aptamers as a vehicle to deliver siRNAs to diseased cells/tissues in a cell type-specific manner. Several examples discussed in this review (for example, covalent aptamer-siRNA chimeras, non-covalent aptamer-connector-siRNA conjugates and aptamer-functionalized nanovectors loaded with siRNAs), provide complementary approaches for combining the power of RNAi with aptamer technology, providing a versatile technology platform for the treatment of various diseases.

Despite substantial progress in aptamer-mediated siRNA delivery, two major efforts are still required for clinical translation: (i) the development of more efficient selection methods to generate new cell-internalizing aptamers with high affinity and (ii) the development of easier conjugation strategies for siRNA joining to aptamers.

## Competing interests

The authors declare that they have no competing interests.

## Authors' contributions

JZ drafted the manuscript. JR revised it and gave final approval of the version to be published. All authors read and approved the final manuscript.
